# Subacute pericardial abscess after aortic valve replacement: a case report

**DOI:** 10.1186/s12879-020-05063-x

**Published:** 2020-05-13

**Authors:** Ilenia Magnani, Alberto Spadotto, Pasquale Paolisso, Alberto Foà, Carlo Savini, Davide Pacini, Carmine Pizzi, Nazzareno Galiè

**Affiliations:** 1 Department of Experimental, Diagnostic and Specialty Medicine-DIMES (Padiglione 23), University of Bologna, Via Giuseppe Massarenti 9, 40138 Bologna, Italy; 2 Cardiac Surgery Unit, Cardio-Thoracic-Vascular Department, S. Orsola Hospital, Alma Mater Studiorum - University of Bologna, Bologna, Italy

**Keywords:** Pericardial abscess, Purulent pericarditis, Constrictive pericarditis, *Escherichia coli*

## Abstract

**Background:**

Purulent pericarditis is an infectious disease, frequently caused by gram-positive bacteria, that is rarely observed in healthy individuals, and is often associated with predisposing conditions.

**Case presentation:**

Here, we present the case of an *Escherichia coli* post-surgical localized purulent pericarditis complicated by transient constrictive pericarditis and its diagnostic and therapeutic management.

**Conclusions:**

Our case report focuses on the importance of imaging-guided treatment of purulent pericardial diseases, in particular on the emerging role of 18 F-labelled 2-fluoro-2-deoxy-D-glucose Positron Emission Tomography/Computed Tomography in pericardial diseases and on the management of transient constrictive pericarditis, often seen after thoracic surgery.

## Background

Purulent pericarditis is defined as the presence of gross pus in the pericardium or microscopic purulence, frequently due to an infective agent; when localized, it becomes a pericardial abscess [1]. Today, in the antibiotic era, purulent pericarditis is rarely observed in healthy adults and the very few reported cases were associated with predisposing factors (immunodeficiency, alcohol abuse, cardiac surgery and chronic renal failure). The main cause is gram-positive bacteria and the typical clinical presentation is acute, with haemodynamic instability [2]. Clinical presentation might be subacute or chronic, usually caused by *Mycobacterium tuberculosis* [3]. However, uncommon cases of subacute purulent pericarditis due to Escherichia (E.) coli infection in patients with predisposing factors have been described [4]. We report a case of *E. coli* pericardial abscess in a patient who underwent cardiac surgery complicated by pericardial constriction.

## Case presentation

In August 2017, a 61-year old man with multiple cardiovascular risk factors – obesity (BMI 30.8 kg/m^2^), hypertension and hypercholesterolemia - underwent aortic valve replacement with a mechanical prosthetic valve for severe aortic stenosis. After surgery, the patient developed a persistent pericardial effusion and was discharged with steroidal therapy. Thereafter, the patient developed type 2 diabetes mellitus. Three months after surgery, the patient complained of exercise dyspnea with no clinical and laboratory signs of active infection. Transthoracic echocardiogram showed persistent pericardial effusion. In presence of post-pericardiotomy syndrome after cardiac surgery, an anti-inflammatory therapy with ibuprofen and colchicine was added on top of chronic steroidal therapy.

In February 2018, he was referred to our hospital for rapidly worsening dyspnoea and lower limb oedema despite pharmacologic therapy. The patient denied fever and had no signs of haemodynamic instability, but the jugular venous pressure was about 12 cm H_2_O. His pulmonary examination was remarkable due to a decrease in breath sounds in both lung bases. Laboratory studies revealed elevated inflammatory markers (White Blood cell Count (WBC) 13,226/mmc, C-Reactive Protein (CRP) 13.91 mg/dl, Procalcitonin (PCT) 1.5 ng/ml).

**Fig. 1:**
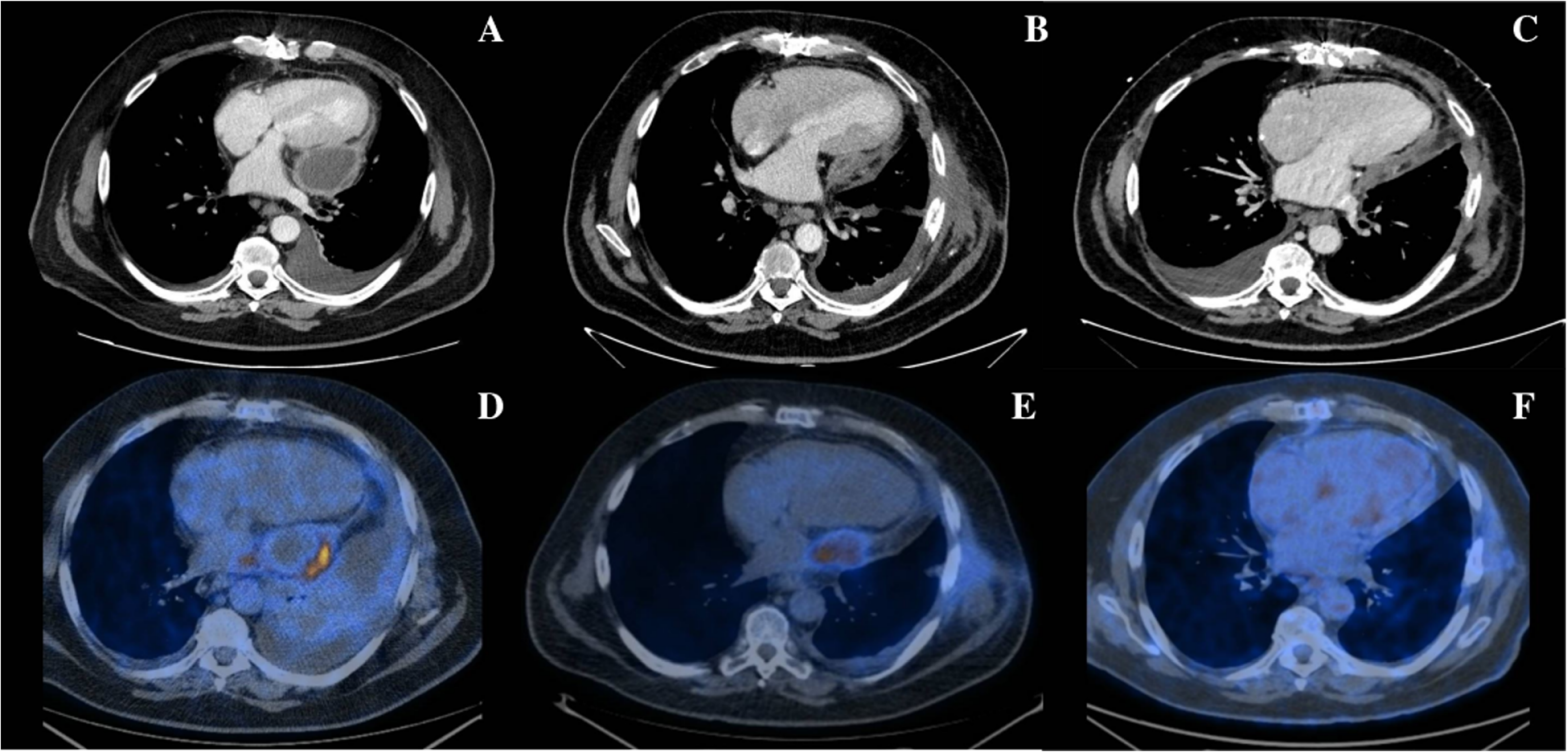
Thoracic computed tomography and 18 F-labeled 2-fluoro-2-deoxy-D-glucose positron emission tomographic/computer tomography imaging. In the upper series of figures, thoracic computed tomography scan sections acquired in February 2018 (**a**), April 2018 (**b**) and October 2018 (**c**), respectively, are shown. (**a**) Severe loculated pericardial effusion localized to the lateral wall of the left ventricle (51 mm) and bilateral pleural effusion. (**b**) Paracardiac egg-shaped fluid collection (38 × 27 mm) with peripheric hypercaptation of the tracer. (**c**) Mild diffuse postero-lateral pericardial effusion (maximal thickness: 11 mm). No evidence of persistence of localized fluid collection. In the lower series, 18 F-labeled 2-fluoro-2-deoxy-D-glucose positron emission tomographic/computer tomography (^18^F-FDG PET/CT) sections acquired in February 2018 (**d**), April 2018 (**e**) and April 2019 (**f**) are presented. **d** Pathological hypercaptation at the walls of the pericardial effusion (SUV max = 11). **e** In comparison with previous ^18^F-FDG PET/CT, the area of tracer hypercaptation in left paracardiac position is reduced in dimension, but stable in intensity (SUV max = 9.6). **d** Normalization of the area of metabolic hypercaptation reported in the previous exams

A transthoracic echocardiogram showed a loculated pericardial effusion with signs of restrictive physiology, normal left ventricular function and no signs of valve dysfunction. Cardiac Computed Tomography (CT) confirmed the echocardiographic findings (Fig. 1a). To exclude an active infection, 18 F-labelled 2-fluoro-2-deoxy-D-glucose Positron Emission Tomography/Computed Tomography (^18^F-FDG PET/CT) was performed and revealed a selective hypercaptation of the tracer at the walls of the pericardial effusion (SUV max = 11) (Fig. 1d). Despite the selective cardiac hypercaptation on ^18^F-FDG PET/CT, urinary and blood cultures, abdominal ultrasound and pancolonscopy were performed and excluded other sources of infection.

Because of the location of the pericardial effusion, video-assisted thoracic surgery pericardial drainage was planned. During the procedure, pleural empyema and a pericardial abscess were revealed, so a conversion to a mini-thoracotomy approach was necessary in order to perform pericardiotomy and pleural decortication. After broad-spectrum antibiotic therapy, an amoxicillin-sensitive *E. coli* was isolated from the pericardial fluid, so targeted intravenous antibiotics were continued for 2 weeks. The patient was discharged with oral amoxicillin/clavulanate therapy aiming to complete a 4-week therapy.

In April 2018, the patient presented to the emergency department complaining of a high fever, worsening dyspnoea and lower limb oedema. An urgent CT scan revealed a paracardiac egg-shaped fluid collection (Fig. 1b). Laboratory tests only showed a mild elevation of inflammatory markers (WBC 11.780/mmc, CRP 5.1 mg/dl, PCT 0.4 ng/ml). Empiric broad-spectrum antibiotic therapy and high dose diuretic therapy were started, and the patient was transferred to the Cardiology Department.

The echocardiogram showed a left postero-lateral fluid collection, without diffuse pericardial effusion. Cardiac magnetic resonance confirmed a non-homogeneous fluid collection, thick walls picking up contrast medium and septal bounce. An ^18^F-FDG PET/CT revealed a persistence of tracer hypercaptation in the left paracardiac position (SUV 9.6 max) (Fig. 1e).

**Fig. 2:**
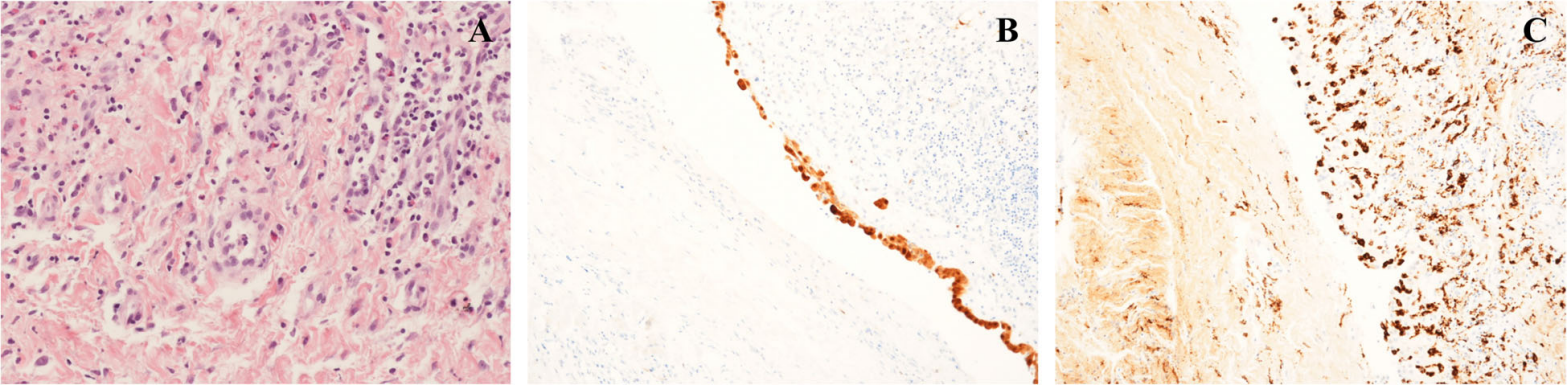
Histological and immunohistochemical analysis. In **a** there is evidence of chronic inflammation with fibrosis, mesothelial hyperplasia and mixed cellular infiltrate rich in macrophages, neutrophils, eosinophils and plasma cells. **b** shows the immunohistochemical analysis for Calretinin that highlights mesothelial cells and their hyperplasia. **c** we can observed the immunohistochemical analysis for CD 68, a specific surface marker of macrophages

Due to the persistence of haemodynamic impairment, the patient underwent surgical excision of the pericardial abscess and partial pericardiectomy through a sternotomy approach. As shown in Fig. 2a the histological analysis demonstrated an underlying chronical inflammation process with fibrosis and mesothelial hyperplasia (Fig. 2b) and inflammatory infiltrate rich in macrophages (Fig. 2c). In the absence of microbial growth at microbiological exams, *E. coli* targeted intravenous antibiotic therapy was continued.

In considerations of the persistence of fluid overload and the new onset of signs of pericardial constriction at echocardiograms, high dose diuretic treatment and colchicine were successfully administered.

The patient was discharged home with long-term oral antibiotic therapy and colchicine.

At the 6- and 12-month evaluations, the patient was asymptomatic, without any symptoms of fluid overload or echocardiographic signs of constrictive physiology. A 6-month CT scan revealed the persistence of mild diffuse pericardial effusion without any sign of pericardial abscess (Fig. 1c). A 12-month 18-FDG-PET did not show any hypercaptation of the tracer in the cardiac area (Fig. 1f).

## Discussion and conclusions

Despite optimized surgical technique and perioperative antibiotic prophylaxis, the incidence of infection after cardiac operations is still high [5]. We have described an unusual case of *E. coli* pericardial abscess in a post-surgical patient. The most common organism that causes purulent pericarditis is *Staphylococcus aureus*. Other frequently isolated bacteria are *Neisseria meningitidis* and *Streptococcus pneumoniae. G*ram-negative rods, *Pseudomonas aeruginosa, Salmonella,* anaerobes, and fungal pathogens are less common [6]. The literature contains few case-reports of purulent pericarditis due to a very uncommon gram-negative bacillus as *E. coli*. Sagrista-Sauleda et al. reported that in 33 patients with purulent pericarditis only two were due to *E. coli* [7] and usually develop in patients with cancer, in particular in aggressive lymphomas [4].

The development of postoperative cardiac infection may be due patient-related and procedure-related factors [8]. In our case report the elements related to the pericardial infection may be obesity and perhaps no optimal hygienic standards to minimize the local bacterial infection.

The clinical presentation in our patient was subacute/chronic and differed from the acute manifestation of the vast majority of purulent pericarditis due to Gram + bacteria. This clinical evolution is probably typical of E.coli pericarditis [4] or might be due to a concomitant immunosuppression condition provoked by chronic antinflammatory therapy - steroidal and non-steroidal therapy - prescribed after cardiac surgery and the diabetic status. Our case report is the first described case of pericardial abscess due to *E. coli* after cardiac surgery. However, it is reported that purulent pericarditis is an unusual complication of thoracic surgery. Pericarditis may be present in the early post-operative period associated with a severe mediastinitis requiring re-operation and drainage. Our patient was discharged with steroidal and non-steroidal anti-inflammatory drugs, demonstrating that the pericarditis was already present, but it was diagnosed as post-pericardiectomy.

In our case, a very careful work-up was essential in guiding the diagnosis and treatment. In particular, ^18^F-FDG PET/CT confirmed the persistence of the inflammation. In heart studies, ^18^F-FDG PET/CT is used to identify areas of viable myocardium and inflamed tissues such as infiltrating inflammatory cells and infectious foci. Its efficacy is due to the ability of ^18^F-FDG PET/CT to dynamically penetrate activated leukocytes, macrophages, and CD4-positive T cells present at the sites of infection. In parallel, recent studies have reported promising data for ^18^F-FDG PET/CT in the diagnosis of infective endocarditis particularly in patients with prosthetic valves and implantable cardiac electronic device infection as well as in the recognition of extracardiac complications [9]. The recent guidelines of the European Society of Cardiology have included ^18^F-FDG PET/CT as a diagnostic tool in the diagnostic algorithm of prosthetic valve endocarditis [10].

A few studies and several case reports have demonstrated that ^18^F-FDG PET/CT imaging provides a measurement of inflamed tissues such as purulent pericarditis. Intense ^18^F-FDG PET/CT uptake has been described in tuberculous pericarditis on ^18^F-FDG PET/CT [11]. Dong et al. reported that ^18^F-FDG PET/CT in patients with purulent pericarditis had a greater intensity of uptake as compared with idiopathic pericarditis. In fact, in our patient’s ^18^F-FDG PET/CT the SUV max was very high [12]. Despite pseudonormalised inflammatory markers, the correct non-invasive imagines helped us in deciding the correct management and the correct follow-up.

Finally, constrictive physiology developed in our patient immediately after pericardiectomy with an urgent need for intravenous diuretics and anti-inflammatory drugs, but almost disappeared at follow-up. In fact, transient constrictive pericarditis is frequently described in the literature after cardiac surgery and is related to the presence of acute inflammation that provokes constrictive symptoms and resolves after standard treatment for acute pericarditis [13].

In conclusion, this case report highlights the importance of the correct microbiological diagnosis in order to best guide antimicrobial therapy and the central role of imaging-guided treatment of purulent pericardial diseases. We also reported the existence of a transient form of constrictive pericarditis, often seen after thoracic surgery, which usually resolves with anti-inflammatory drugs.

## Data Availability

Not applicable.

## Abbreviations

*E. coli*: *Escherichia coli*
WBC: White Blood cell Count
CRP: C-Reactive Protein
PCT: Procalcitonin
CT: Computed Tomography
^18^F-FDG PET/CT: 18 F-labelled 2-fluoro-2-deoxy-D-glucose Positron Emission Tomography/Computed Tomography
